# Supplementary figures and images for: Improving the prediction of potato yield gaps: Solanum-model parameterization and evaluation in southwestern China
Source: PLoS One. 2025 Aug 7;20(8):e0328675. doi: 10.1371/journal.pone.0328675 (PMC12331027; doi:10.1371/journal.pone.0328675)

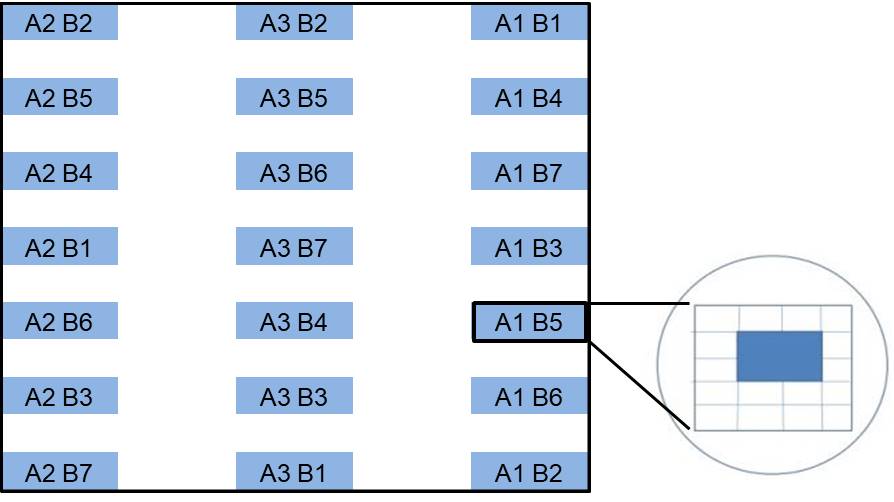

Supplement: S1 Fig — (JPG) [file pone.0328675.s001.jpg]
