# Supplementary material for: Improving the prediction of potato yield gaps: Solanum-model parameterization and evaluation in southwestern China
Source: PLoS One. 2025 Aug 7;20(8):e0328675. doi: 10.1371/journal.pone.0328675 (PMC12331027; doi:10.1371/journal.pone.0328675)
Supplement: S1 Table — (DOCX) [file pone.0328675.s002.docx]

**Table S1: Soil Information of the three experimental sites Yema, Songming, and Chaotie.**

|  | pＨ | Organic Matter | Total Nitrogen | Total Phosphorus | Total Potassium | Hydrolyzable Nitrogen | Available Phosphorus | Available Potassium |
| --- | --- | --- | --- | --- | --- | --- | --- | --- |
| Yema | 5.4 | 70.75 | 3.08 | 1.88 | 12.11 | 133.98 | 26.26 | 148.15 |
| Songming | 5.77 | 39.58 | 6.85 | 2.36 | 18.22 | 132.51 | 76.67 | 228.68 |
| Chaotie | 6.2 | 23.41 | 4.04 | 0.74 | 24.77 | 103.08 | 31.39 | 127.18 |
