# Supplementary material for: Improving the prediction of potato yield gaps: Solanum-model parameterization and evaluation in southwestern China
Source: PLoS One. 2025 Aug 7;20(8):e0328675. doi: 10.1371/journal.pone.0328675 (PMC12331027; doi:10.1371/journal.pone.0328675)
Supplement: Attachment — Submitted filename: Response to reviewers.doc [file pone.0328675.s004.doc]

**Responses to Reviewers**

**Replies to Reviewer #1 and revisions made:**

**Comment: 1.** Introduction should be written again. It is very short and most of the information is related to geography and yield. It is better if author include more review about the different models being used in the world and how they are beneficial for farmers. Hypothesis is missing. Rephrase the objective of the studies.

**Response**: We sincerely thank the reviewer for the constructive feedback on the Introduction section. As suggested, we have thoroughly revised the Introduction to improve its depth and clarity. Specifically, we have: added a comprehensive review of globally used crop simulation models (e.g., APSIM, DSSAT, STICS, EPIC, and POMME), highlighting their relevance and benefits to farmers in terms of yield optimization, resource management, and climate resilience. Clearly stated the hypothesis of the study, focusing on the role of biotic and abiotic factors in contributing to potato yield gaps in Yunnan. Rephrased and clarified the objective of the study to better reflect the goals of quantifying potential yield, identifying limiting factors, and evaluating the Solanum model's applicability for enhancing yield outcomes. Now, we believe these revisions have significantly strengthened the scientific context and relevance of the study.

**Comment: 2.** Materials and methods:

No weather and soils information are given about the studied locations.

**Response:** Thank you for pointing out that weather information is misssing. In response to your valuable suggestion, we have now included detailed **meteorological data**—covering temperature, rainfall, solar radiation, and sunshine hours—for each cropping season and experimental location in **Table 2** of the revised manuscript. Additionally, we have provided the **soil characteristics** of the three experimental sites (Yema, Songming, and Chaotie) in **Supplementary Table S1**. These additions enhance the environmental context of the study and support the interpretation of the model outputs and yield results.

**Comment: 3:** Ten years old research During the growth periods of 2013-2015.

**Response:** We acknowledge the reviewer’s observation regarding the period of the field experiments (2013–2015). While the data were collected nearly a decade ago, they remain highly relevant for several reasons:

1. Baseline Model Calibration: The purpose of the study is to evaluate the Solanum model's parameterization under well-documented field conditions. The historical dataset provides strong and detailed agronomic, phenological, and environmental information essential for validating and improving the model's accuracy.
2. Climatic Stability of the Region: Yunnan Province exhibits relatively stable climatic patterns over the years, particularly in terms of temperature and altitude-dependent cropping systems. Thus, the findings remain applicable to current cultivation practices.
3. Limited Shifts in Cultivar Use: The cultivars studied are still widely grown or serve as the genetic basis for more recent breeding lines, making the analysis and conclusions still pertinent to ongoing production systems.
4. Model Utility Over Time: Since crop simulation models like Solanum are meant to provide generalizable insights across years and conditions, their calibration using historical datasets enhances future projections and current applicability.We agree that future studies should aim to include more recent datasets and are planning to validate the model under updated climate scenarios and recent field data in ongoing research.

**Comment: 4:** Line 94-96: The total growth period 94 from planting to harvesting for the early spring, spring and autumn seasons, spanned from early January to the end of 95 May, mid-March to end-of-September, and mid-August to mid-December for early-spring, spring, and autumn seasons, 96 respectively. This sentence should be written again.

**Response:** Thank you for the suggestion. We have revised the sentence and marked in blue color. “The complete growth cycles for the three cultivation seasons were characterized as follows: early spring potatoes (January-May, ~150 days), spring potatoes (March-September, ~180 days), and autumn potatoes (August-December, ~120 days). This distinct seasonal partitioning reflects Yunnan's unique capacity for year-round potato production enabled by its vertical climatic zonation.”.

**Comment: 5:** No tables are provided in the PDF file.

**Response:** We have included tables in the PDF

**Comment: 6:** The paper is not written according the PLOS one standard.

**Response:** Thank you for suggestion. We have thoroughly revised the manuscript and written according to the PLOS one standard.

**Comment: 6**: Authors is not clear about the results.

**Response:** Thank you for your valuable feedback. We have carefully reviewed the manuscript and thoroughly revised it, and ensured that the presentation of our results is clear and precise. We believe that the revised version addresses the concerns raised, and we have made efforts to clarify our findings and avoid any uncertainty. We hope that the revised manuscript reveals our clarity on the results and provides a more clear narrative. If further clarification is needed, we are happy to make additional revisions." Thank you for the comments.

**Comment: 6**: Discussion is very weak.

**Response:** Thank you for your constructive feedback regarding the discussion section. We appreciate your insights, we have thoroughly revised the discussion to better interpret the results and relate them to the existing literature. We have also provided additional context and analysis to strengthen our conclusions and clarify the implications of our findings. We believe that these revisions enhance the overall quality of the discussion, and we hope the updated section now meets the expectations. Should you have further suggestions or points for improvement, we are happy to address them."
